# PFO morphology for evaluation of c-TCD and c-TTE RLS grades

**DOI:** 10.1186/s40001-022-00855-0

**Published:** 2022-11-03

**Authors:** Jiali Tian, Xiaobo Chen

**Affiliations:** Ultrasound Department, The Fifth Affiliated Hospital of Sun Yat-Sen University, Zhuhai, 519000 China

**Keywords:** PFO, Contrast-transthoracic echocardiography, Contrast-transcranial Doppler ultrasonography, Transesophageal echocardiography, IAS mobility

## Abstract

**Purpose:**

The purpose of this study was to observe the morphologic characteristics of patent foramen ovale (PFO) by transesophageal echocardiography (TEE), and to analyze its correlation with right-to-left shunt (RLS) of contrast-transthoracic echocardiography (c-TTE) and contrast-transcranial Doppler ultrasonography (c-TCD).

**Methods:**

124 patients with PFO were divided into four groups according to the morphological characteristics of PFO. RLS grade of each group PFO with c-TTE and c-TCD in resting and Valsalva manoeuvre was measured. Anatomical structures influencing RLS grade were analyzed statistically through multivariate logistic analyses and predictive models.

**Results:**

The 124 cases of PFO were divided into four groups: 55 cases (44.4%) with smooth uniform tubular tunnel (SUT), 21 cases (16.9%) with granule uniform tubular tunnel (GUT), 23 cases (18.5%) of right funnelform, 25 cases (20.2%) of left funnelform. Between group comparisons and multivariate logistic analyses revealed that PFO morphotype and interatrial septum(IAS) mobility were influencing factors of RLS degree. During Valsalva, the probability of c-TCD RLS ≥ 2 for the right funnelform PFO was 13.428 times that of the GUT, one unit increase in IAS mobility increased the probability of c-TCD RLS ≥ 2 by a factor of 2.029, model predicted c-TCD RLS ≥ 2 with 78.1% sensitivity and 94.7% specificity; During Valsalva, the probability of c-TCD RLS ≥ 2 for the SUT PFO was 4.244 times that of the GUT, one unit increase in IAS mobility increased the probability of c-TTE RLS ≥ 2 by a factor of 2.392, model predicted c-TTE RLS ≥ 2 with 80.2% sensitivity and 87.9% specificity.

**Conclusions:**

Studies have shown that the morphological structure of PFO is an influencing factor of RLS, and TEE can observe the specific morphological characteristics of PFO, which can further predict the level of RLS, help predict the occurrence of Cryptogenic stroke (CS). The above provides more evidences and surgical options for Interventional device closure indications.

## Introduction

CS is an unexplained ischemic stroke in 30–40% of patients after excluding cerebrovascular disease, cardioembolic stroke and other vascular causes. Previous studies have shown that about half of patients under 55 with CS are associated with paradoxical embolism from RLS through the PFO [1–3]. Simple PFO is defined as tunnel length (the distance between septum secundum and septum primum overlapping) less than 8 mm, and the thickness of septum secundum is less than 6 mm, without atrial septal aneurysm (ASA) and excessively long Eustachian valve(EV) or Chiari Network; Complex PFO is the presence of ASA, tunnel length > 8 mm, with Lipomatous hypertrophy of the atrial septum (LHAS), Chiari network and Eustachian valve, multifenestrated ASA, and anatomically distorted PFO [4, 5]. The structure of the PFO is similar to that of a valve, since the pressure of the left atrial is higher than that of the right atrium, it is generally closed. When the right atrial pressure is higher than the left atrial pressure, the primary septum is pushed open and the foramen ovale reopens, resulting in RLS. The higher the RLS, the higher the incidence of paradoxical embolism [6]. Previous studies have shown that large size PFOs, long-tunnel PFOs, atrial septal aneurysm, hypermobile interatrial septum, prominent Eustachian valve and Chiari network are risk factors for CS [1, 7, 8]. Interventional device closure is beneficial to prevent embolism when the PFO is complicated by paradoxical embolism or the size of RLS of the PFO is substantial [9, 10]. At present, the degree of RLS of the PFO is judged by contrast-transthoracic echocardiography or contrast-transcranial Doppler ultrasonography. To assess the presence of PFO and the grade of shunt, it is complicated for patients to complete TEE and c-TTE examinations before surgery.

The morphological classification of PFO and the RLS degree of corresponding types have not been investigated in previous studies. In this study, the left atrial height, right atrial height and tunnel characteristics of PFO were observed and classified by TEE, and the correlation between various types and c-TCD or c-TTE RLS was studied. The RLS grade was predicted by PFO morphology type to guide interventional closure.

## Methods

From January 2020 to December 2021, we enrolled 124 patients, with PFO, Sign informed consent. Those who met the inclusion and exclusion criteria were examined by TEE, c-TTE and c-TCD. Morphological characteristics and tunnel characteristics of PFO in resting and Valsalva manoeuvre were observed by TEE, Intra-atrial parameters and other indicators and RLS levels were measured and recorded.

### Inclusion criteria

1. Patients with unexplained migraine, paradoxical embolism or clinically suspected PFO;
2. Transthoracic echocardiography found suspicious PFO, and further examination was required;
3. Subjects could accept TEE examination under psychological and physiological conditions.

### Exclusion criteria

1. Severe pulmonary hypertension;
2. Severe emphysema;
3. Respiratory failure;
4. Severe anemia;
5. Acidosis and severe heart and kidney dysfunction;
6. Acute coronary syndrome;
7. The subjects cooperated poorly and were unable to complete the Valsalva manœuvre;
8. With atrial septal aneurysm.
9. Anesthetic allergy;
10. TEE contraindications;
11. Subjects were unable to undergo TEE, c-TTE and c-TCD examinations after evaluation by clinicians and sonographers.

### Subject’s informed consent

Investigators should fully inform subjects who met the inclusion and exclusion criteria, or their legal representatives, of all relevant aspects of the study before proceeding with any prescribed procedures. The subject's consent to the study should be recorded. (2) The investigator should ensure that the subject signed and date the informed consent form in person. Any manipulation should be done after completing the informed consent procedure.

### Baseline characteristics

Record the gender, age, BMI, left ventricular EF value, underlying diseases (hypertension, coronary heart disease, diabetes, hyperlipidemia), smoking history, drinking history, and clinical symptoms of subjects who met the inclusion and exclusion criteria (Unexplained stroke, migraine, dizziness, history of syncope, chest tightness).

### TEE procedure

**Fig. 1:**
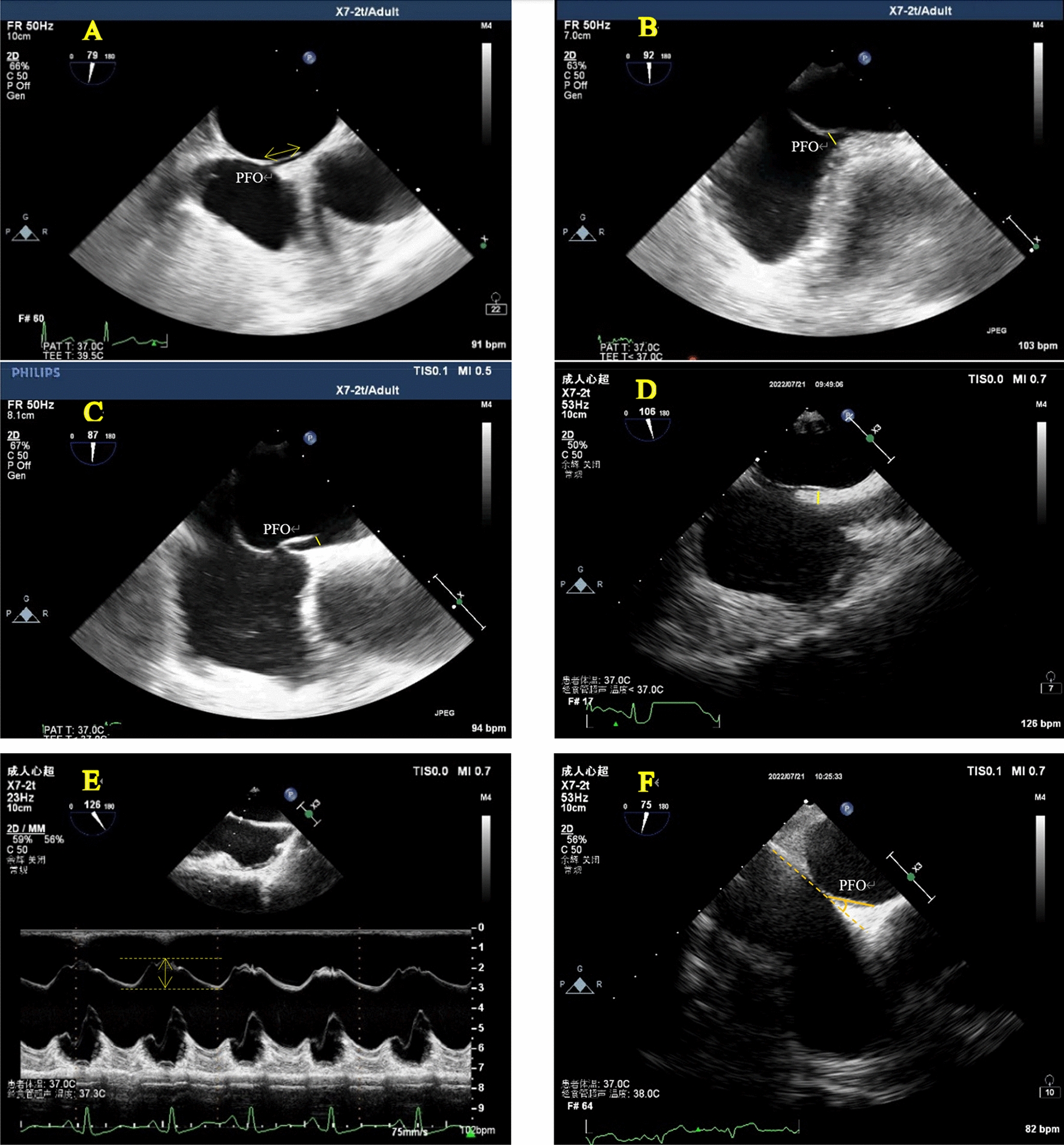
**A**: PFO tunnel length. **B** Right atrial height of the PFO. **C** Left atrial height of the PFO. **D** septum secundum thickness. **E** IAS mobility distance. **F** PFO angle

The subjects fasted for 6–8 h before TEE, removed the oral dentures, and performed local anesthesia on the throat surface with lidocaine hydrochloride mortar (Handan Kangye Pharmaceutical, National Medicine Approval No. H13021217). After being fully anesthetized for about 15 min, a bite protector was placed in the oral cavity of the subject. Use Philips IE Elite Color Doppler Ultrasound or Philips EPIQ 7C Color Doppler Ultrasound and Transesophageal Ultrasound Probe X7-2t, frequency 4–7 MHz. During the examination, the subjects were placed in the left lateral decubitus position, the probe was placed in the middle of the esophagus, and the views of the double atrium, superior vena cava and inferior vena cava were clearly displayed, and the angle was fine-tuned to show the complete picture of the separation of the septum secundum and septum primum. Two-dimensional and color Doppler videos of at least 3 cardiac cycles were obtained. On-machine image analysis was performed by two cardiac ultrasound specialists above the attending level. The following parameters were observed and recorded: right atrial height, left atrial height, tunnel length, septum secundum thickness, IAS mobility, PFO angle and transseptal blood flow characteristics of the PFO, as shown in Fig. 1. The length of the PFO tunnel was the maximum overlapping distance between the septum secundum and septum primum; IAS mobility distance: the measurement line was placed in the middle of the atrial septum to measure the total offset distance of the left and right atrium; PFO angle: the mid-esophageal view showed and measured the angle of the inferior vena cava and the foramen ovale flap.

### c-TTE method

Agitated saline contrast (ASC) was be injected, while the subjects were in a resting state. In addition, after the right atrium was completely visualized, the amount of microbubbles in the left heart was observed within 3–5 cardiac cycles. Repeat the above steps after the left heart microbubbles disappeared, and continue to observe the number of microbubbles in the left heart cavity [11, 12]. During the process, the subjects felt discomfort and terminated the examination immediately.

### c-TCD method

The RLS grade was assessed using a transcranial Doppler ultrasound system (DWL, Germany) with a bitemporal 2 MHz probe. The subject was in a semi-recumbent position, and the indwelling needle was placed in the subject's forearm superficial vein and connected to a three-way tube. The contrast agent was prepared by mixing 9 ml of normal saline and 1 ml air. Rapid bolus injection was performed, and the microbubbles signal was monitored within 10 s. Repeat the above steps in the Valsalva manoeuvre.

### Diagnostic criteria

1. Diagnosis of PFO by TEE: separation between the septum secundum and septum primum, visible between the primary and secondary septa blood flow signal [13].
2. The c-TTE grading standard refers to Agitated Saline Contrast Echocardiography in the Identification of Intra-and Extracardiac Shunts: Connecting the Dots [12]: according to the maximum number of microbubbles appearing in the left heart cavity of a static single frame image, the degree of shunt is divided into 4 grades. Grade 0: the number of microbubbles in the left heart cavity is 0; Grade 1: the number of microbubbles is 1–9; Grade 2: the number of microbubbles is 10–30; Grade 3: the number of microbubbles is more than 30.
3. The c-TCD grading standard refers to the 2000 Detection of Right-to-Left Shunt with Ultrasound Contrast Agent and Transcranial Doppler Sonography [14]. According to the number of microbubbles, it is divided into four levels: Grade 0: no microbubbles are detected; Grade 1: 1–10 microbubbles; Grade 2: more than 10 microbubbles, non-curtain; Grade 3: embolic signal Curtain or shower type.
4. RoEF score: Refer to the CS risk stratification score for patients with PFO created by Dr. Ken et al. [15]. No history of hypertension (1 point), no history of diabetes (1 point), no history of stroke or TIA (1 point), no smoking history (1 point), cortical infarction (1 point), age 18–29 years (5 points), age 30–39 years (4 points), age 40–49 years (3 points), age 50–59 years (2 points), age 60–69 years (1 point), age ≥ 70 years (0 points).

### Four PFO anatomical features

**Fig. 2:**
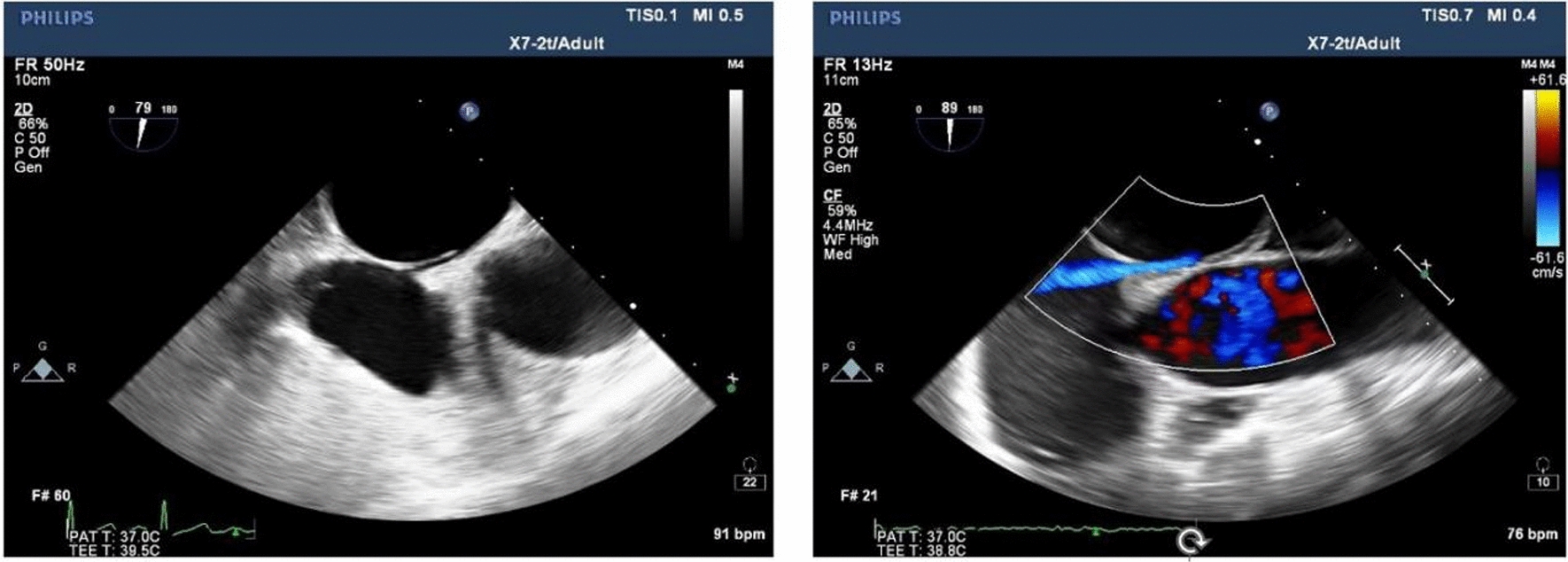
SUT

**Fig. 3:**
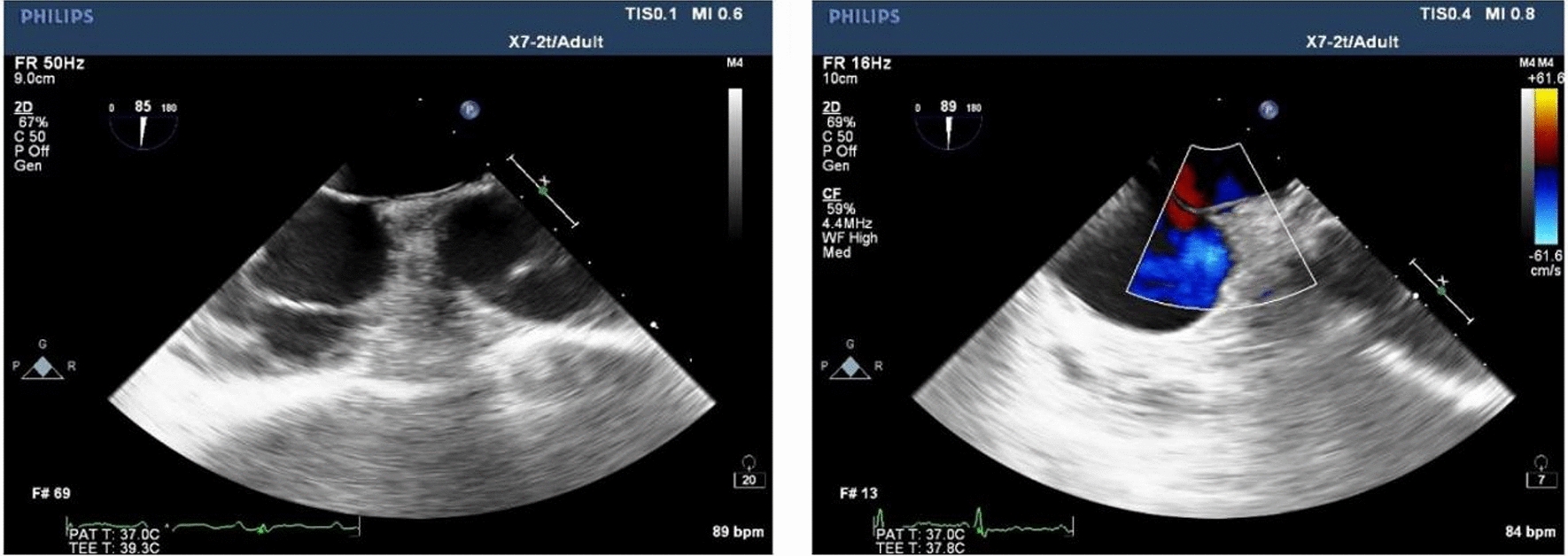
GUT

**Fig. 4:**
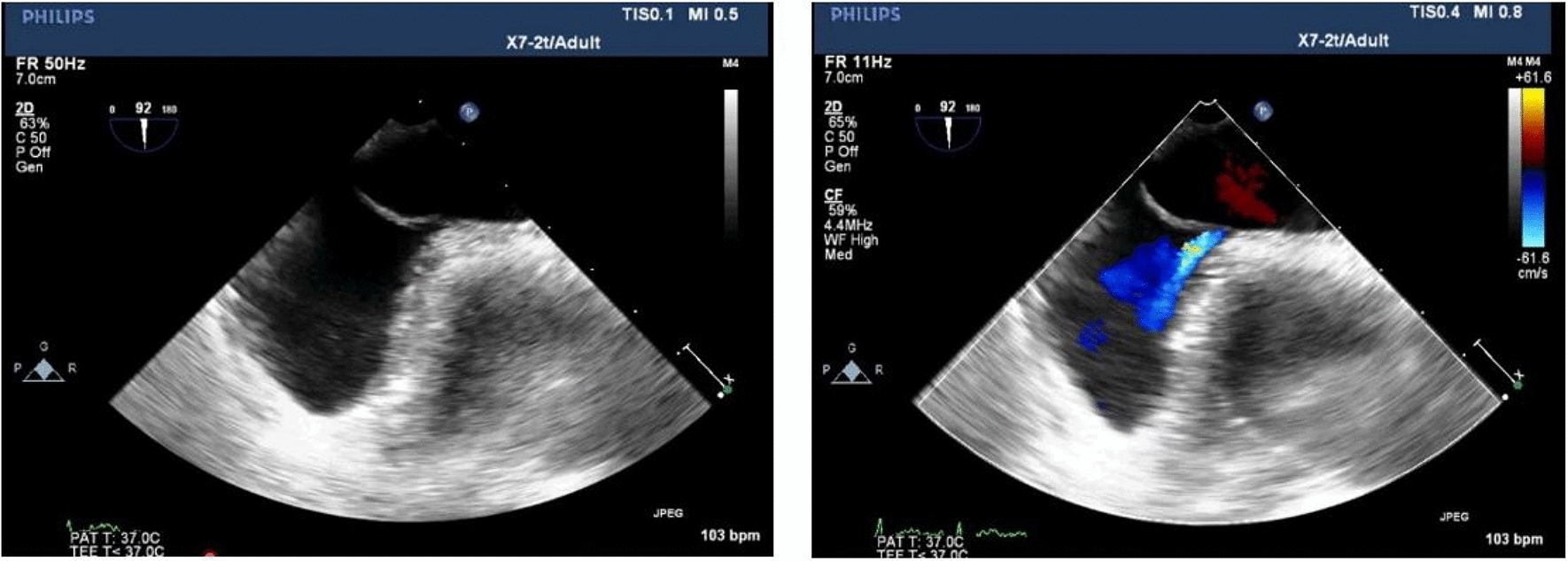
Right funnelform

**Fig. 5:**
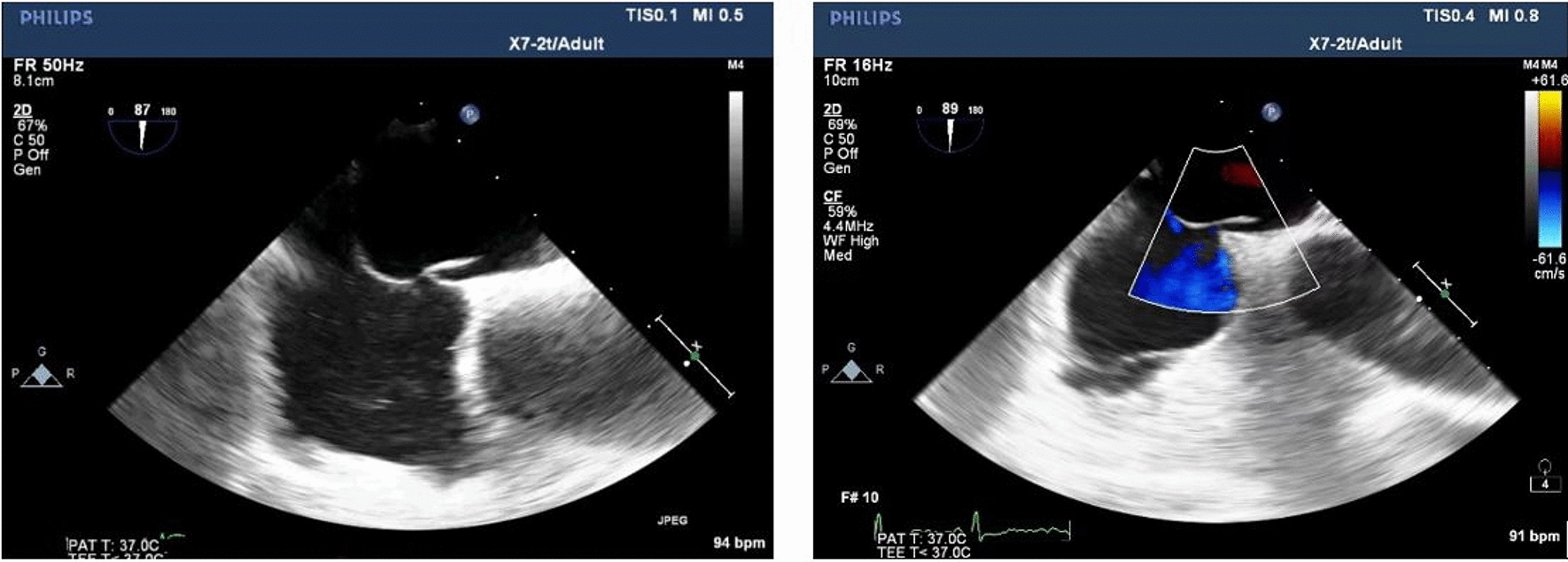
Left funnelform

Referring to the study of Jun Tanaka et al. [16], the anatomical morphology of PFO observed by TEE in this study was divided into the following four types: (1) SUT: the height of the right atrial side of the PFO tunnel was similar to that of the left atrium side, there was no obvious bulge of fat particles in the tunnel, and the inside of the tunnel showed a uniform strip-shaped transseptal blood flow signal (Fig. 2); (2) GUT: fat-like thickening of septum secundum, adipose tissue-like bulges could be seen in the tunnel, causing local adhesion of septum primum and septum secundum, the tunnel was segmented and the inner diameter was generally less than 2.0 mm, and the interior was mainly dot-shaped or short-rod-shaped blood flow signals (Fig. 3); (3) right funnelform: the primary septum primum and septum secundum at the tunnel exit were not clearly separated, and the inner diameter was mostly less than 2.0 mm. The septum primum and septum secundum at the entrance were obviously separated, and the difference from the inner diameter at the exit was greater than 2.0 mm, which was narrow strip and mainly bright blood flow signals (Fig. 4); (4) the left funnelform was the opposite of the right funnelform. The inside of the tunnel was mostly dim blood flow signal, and the blood flow at the entrance was brighter (Fig. 5).

### Statistical analysis

SPSS26.0 was used for statistical analysis of the data. The measurement data were tested by normality. The data conforming to the normal distribution were expressed by mean ± standard deviation, and the inter group comparison was performed by independent sample *T* test; The data that did not conform to the normal distribution were expressed by the median (quartile), and the Mann–Whitney *U* test was used for comparison between groups. Categorical enumeration data are expressed by the number of cases (percentage). The chi-square test was used for comparison between groups. Logistic regression was used to analyze the factors affecting the shunt classification of c-TCD and c-TTE. The diagnostic value of the model was analyzed by ROC curve, *P* < 0.05 was statistically significant.

## Result

### General situation of the research population

A total of 124 subjects were included in this study, with an average age of 49.38 ± 13.32, 58 males (46.77%) and 66 females (53.23%). Four types of PFO: 55 cases of SUT (44.35%), 21 cases of GUT (16.93%), 23 cases of right funnelform (18.55%), and 25 cases of left funnelform (20.16%). The general basic date of the four types of PFO is shown in Table 1.

**Table 1:** General basic data of four types of PFO

Patient characteristics	SUT	GUT	Right funnelform	Left funnelform	*F*	*P*
Age	48.27 ± 12.67	50.67 ± 14.39	52.04 ± 12.84	48.28 ± 14.56	0.549	0.650
Gender: (Male: Female)	(20:35)	(12:9)	(14:9)	(12:13)		0.161
BMI	23.96 ± 3.43	23.59 ± 3.08	22.84 ± 3.38	23.71 ± 3.50	0.593	0.621
Left ventricular EF value (%)	70.01 ± 4.29	69.09 ± 4.07	69.70 ± 4.14	69.88 ± 4.52	0.244	0.865
Hypertension	19 (34.55%)	7 (33.33%)	7 (30.43%)	4 (16.00%)		0.392
Atrial fibrillation	2(3.64%)	2 (9.53%)	0	0		0.231
Diabetes	3 (5.45%)	1 (4.76%)	4 (17.39%)	2 (8.00%)		0.319
Hyperlipidemia	17 (30.91%)	7 (33.33%)	4(17.39%)	4 (16.00%)		0.324
Smoking	9 (16.36%)	6 (28.57%)	5 (21.74%)	1 (4.00%)		0.146
Drinking	5 (9.09)	0	2(8.70%)	0		0.223
Symptom						
Unexplained stroke	9 (16.36%)	4(19.05%)	6 (26.09%)	5 (20.00%)		0.803
Migraine	21 (38.18%)	4 (19.05%)	6 (26.09%)	9 (36.00%)		0.373
Dizziness/syncope	22 (40.00%)	9 (42.86%)	11 (47.83%)	8 (32.00%)		0.725
Chest tightness	3 (5.45%)	4 (19.05%)	0	3 (12.00%)		0.091
Head CT/MRI (ischemic focus: normal)	(15:40)	(4:17)	(3:20)	(8:17)		0.395

### Analysis of factors affecting c-TCD shunt classification in resting state

#### Comparison of clinical data among different c-TCD shunt classification groups

The comparison results of clinical data of different c-TCD shunt classification groups are shown in Table 2. The age of the subjects in the c-TCD RLS ≥ 2 group was significantly higher than that in the c-TCD RLS < 2 group, and the difference was statistically significant (P < 0.05). There were no significant differences in BMI, left ventricular EF%, PFO angle, and RoEP score between the two groups (P > 0.05). The right atrial height and left atrial height of the PFO in the c-TCD RLS ≥ 2 group were significantly higher than those in the c-TCD RLS < 2 group, The PFO tunnel length of the c-TCD RLS ≥ 2 group was lower than that of the c-TCD RLS < 2 group, and the difference was statistically significant (P < 0.05), There was no statistically significant difference in septum secundum thickness and IAS mobility distance between the two groups (P > 0.05).

**Table 2:** Comparison of clinical data of c-TCD RLS classification between two groups

Characteristics	c-TCD RLS classification		*t/Z/χ*^*2*^	*P*
	RLS < 2 (*n* = 100)	RLS ≥ 2 (*n* = 24)		
Gender(Male:Female)	50:50	8:16	2.160	0.142
Age	48.13 ± 12.89	54.58 ± 14.08	2.163	0.032
BMI	23.76 ± 3.42	23.13 ± 3.13	0.826	0.410
Left ventricular EF% value	69.86 ± 4.31	69.42 ± 3.96	0.459	0.647
Hypertension	29 (29.0)	7 (29.2)	0.000	0.987
Diabetes	9 (9.0)	1 (4.2)	0.132	0.716
Hyperlipidemia	29 (29.0)	4 (16.7)	1.507	0.220
Unexplained stroke	20 (20.0)	4 (16.7)	0.007	0.933
Smoking	19 (19.0)	2 (8.3)	0.899	0.343
Drinking	5 (5.0)	2 (8.3)	0.020	0.886
Migraine	30 (30.0)	10 (41.7)	1.206	0.272
Dizziness/syncope	42 (42.0)	8 (33.3)	0.604	0.437
Chest tightness	8 (8.0)	2 (8.3)	0.000	1.000
Right atrial height of the PFO	1.50 (1.10, 2.60)	2.90 (1.50, 3.28)	−3.768	< 0.001
Left atrial height of the PFO	1.70 (1.20, 2.75)	2.45 (1.50, 3.18)	−2.287	0.022
PFO tunnel length	8.55 (6.90, 10.20)	6.80 (5.73, 8.45)	−2.980	0.003
septum secundum thickness	4.80 (3.83, 6.20)	4.20 (3.18, 5.50)	−1.307	0.191
PFO Types			4.482	0.206
SUT	20 (20.0)	1 (4.2)		
GUT	42 (42.0)	13 (54.2)		
Right funnelform	17 (17.0)	6 (25.0)		
Left funnelform	21 (21.0)	4 (16.7)		
IAS mobility distance	3.00 (2.00,5.00)	4.50 (3.00,6.00)	−1.628	0.104
PFO angle	20.25 ± 7.19	20.29 ± 7.21	0.025	0.980
RoEP score	6.09 ± 1.96	5.54 ± 1.82	1.250	0.214

### Analysis of factors affecting c-TCD RLS grading in Valsalva manoeuvre

#### Comparison of clinical data of c-TCD RLS classification between two groups

Comparison of clinical data of c-TCD RLS classification between two groups are shown in Table 3. There was a statistically significant difference in the number of PFO morphological cases (*P* < 0.05).

**Table 3:** Comparison of clinical data of c-TCD RLS grading between two groups

Characteristics	c-TCD RLS classification		*t/Z/χ*^*2*^	*P*
	RLS < 2 (*n* = 19)	RLS ≥ 2 (*n* = 105)		
Gender(Male:Female)	11:8	47:58	1.115	0.291
Age	47.84 ± 13.88	49.66 ± 13.27	0.545	0.587
BMI	23.99 ± 3.54	23.58 ± 3.35	0.495	0.621
Left ventricular EF% value	70.32 ± 3.97	69.68 ± 4.29	0.604	0.547
Hypertension	7 (36.8)	29 (27.6)	0.664	0.415
Diabetes	1 (5.3)	9 (8.6)	0.001	0.976
Hyperlipidemia	8 (42.1)	25 (23.8)	2.757	0.097
Unexplained stroke	6 (31.6)	18 (17.1)	1.323	0.250
Smoking	6 (31.6)	15 (14.3)	2.301	0.129
Drinking	0 (0.0)	7 (6.7)	0.383	0.536
Migraine	3 (15.8)	37 (35.2)	2.785	0.095
Dizziness/syncope	9 (47.4)	41 (39.0)	0.463	0.496
Chest tightness	1 (5.3)	9 (8.6)	0.001	0.976
Right atrial height of the PFO	1.1 (0.8,1.2)	2.1 (1.3,2.8)	−4.435	< 0.001
Left atrial height of the PFO	1.3(1.1,1.4)	2.1 (1.25,3)	−2.737	0.006
PFO tunnel length	8.9 (6.5,11.5)	7.9 (6.5,9.7)	−1.332	0.183
septum secundum thickness	9.3 (4.9,10.5)	4.3 (3.75,5.5)	−3.707	< 0.001
PFO Types			20.262	< 0.001
SUT	11 (57.9)	10 (9.5)		
GUT	5 (26.3)	50 (47.6)		
Right funnelform	1 (5.3)	22 (21.0)		
Left funnelform	2 (10.5)	23 (21.9)		
IAS mobility distance	2.00 (1.00,2.00)	5.00 (2.00,6.00)	−4.792	< 0.001
PFO angle	18.95 ± 6.82	20.50 ± 7.23	0.866	0.388
RoEP score	5.68 ± 1.95	6.04 ± 1.94	0.733	0.465

The Mann–Whitney *U* test results showed right atrial height of the PFO, left atrial height of the PFO, and IAS mobility distance in the c-TCD RLS ≥ 2 group were significantly higher than those in the c-TCD RLS < 2 group, and the septum secundum thickness was significantly lower than that in the c-TCD RLS < 2 group, and the differences were statistically significant (*P* < 0.05). There was no significant difference in the PFO tunnel length between the two groups (*P* > 0.05).

#### Logistic regression analysis of the influence of c-TCD RLS classification

Logistic regression analysis of the influence of c-TCD RLS classification are shown in Table 4. The subjects were instructed to perform Valsalva maneuvers, and a binary logistic regression model was established with c-TCD RLS grade ≥ 2 as the dependent variable, and IAS mobility distance and PFO morphology as independent variables. The results showed that the probability of c-TCD RLS ≥ 2 for the right funnelform PFO is 13.428 times that of the GUT, one unit increase in IAS mobility increased the probability of c-TCD RLS ≥ 2 by a factor of 2.029.

**Table 4:** Logistic regression analysis of the influence of c-TCD RLS classification

Factors	*B*	*SE*	*Waldχ*^*2*^	*P*	*OR* (95%* CI*)
IAS mobility distance	0.707	0.229	9.556	0.002	2.029 (1.296 ~ 3.177)
PFO Types	–	–	7.758	0.051	–
GUT	–	–	–	–	1.000
SUT	1.252	0.704	3.163	0.075	3.498 (0.880 ~ 13.901)
Right funnelform	2.597	1.181	4.839	0.028	13.428 (1.327 ~ 135.860)
Left funnelform	1.732	0.933	3.449	0.063	5.655 (0.909 ~ 35.192)
Constant	-1.259	0.603	4.365	0.037	–

#### Diagnostic value of the model for c-TCD RLS classification in Valsalva

**Fig. 6:**
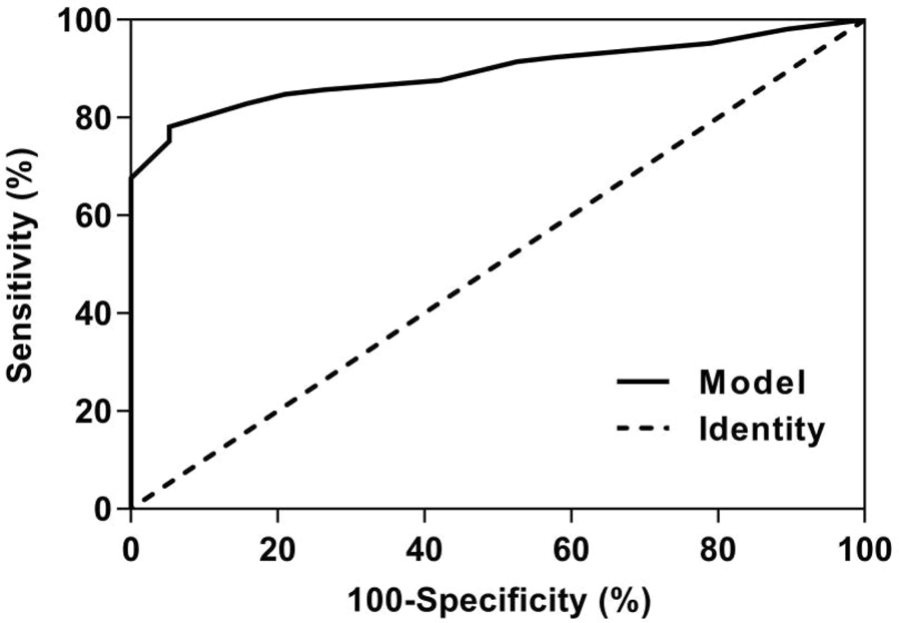
ROC curve analyses of anatomic features of PFO in predicting c-TCD RLS grades

ROC curve analyses of anatomic features of PFO in predicting c-TCD RLS are provided in Fig. 6.The ROC curve showed that the area under the curve for the model to diagnose c-TCD RLS ≥ 2 grade was 0.894 (0.839 ~ 0.950), the corresponding threshold was 0.83597, the sensitivity was 78.1%, and the specificity was 94.7%.

### Analysis of factors affecting c-TTE shunt classification in resting state

#### Comparison of clinical data among different c-TTE shunt classification groups

The comparison results of clinical data of different c-TTE shunt classification groups are shown in Table 5. There was a statistically significant difference in PFO morphology between the two groups (*P* < 0.05). The right atrial height and IAS mobility in the c-TTE RLS ≥ 2 group were significantly higher than those in the c-TTE RLS < 2 group, The PFO tunnel length of the c-TTE RLS ≥ 2 group was lower than that of the c-TTE RLS < 2 group, and the difference was statistically significant (*P* < 0.05).

**Table 5:** Comparison of clinical data of c-TTE RLS classification between two groups

Characteristics	c-TTE RLS classification		*t/Z/χ*^*2*^	*P*
	RLS < 2 (*n* = 104)	RLS ≥ 2 (*n* = 20)		
Gender(Male:Female)	51:53	7:13	1.328	0.249
Age	48.62 ± 13.10	53.35 ± 14.08	1.463	0.146
BMI	23.79 ± 3.40	22.87 ± 3.15	1.121	0.264
Left ventricular EF% value	69.71 ± 4.41	70.10 ± 3.23	0.374	0.709
Hypertension	29 (27.9)	7(35.0)	0.412	0.521
Diabetes	9 (8.7)	1(5.0)	0.010	0.919
Hyperlipidemia	29 (27.9)	4 (20.0)	0.534	0.465
Unexplained stroke	20 (19.2)	4 (20.0)	0.000	1.000
Smoking	19 (18.3)	2 (10.0)	0.333	0.564
Drinking	5 (4.8)	2 (10.0)	0.154	0.695
Migraine	33 (31.7)	7 (35.0)	0.082	0.775
Dizziness/syncope	41 (39.4)	9 (45.0)	0.217	0.641
Chest tightness	10 (9.6)	0 (0.0)	0.996	0.318
Right atrial height of the PFO	1.40 (1.10, 2.60)	2.90 (2.10, 3.45)	−4.350	< 0.001
Left atrial height of the PFO	1.80 (1.20, 2.98)	2.15 (1.33, 2.70)	−0.997	0.319
PFO tunnel length	8.00 (6.90, 10.18)	7.15 (5.73, 8.90)	−2.253	0.024
septum secundum thickness	4.85 (3.90, 6.20)	4.00 (3.33, 5.25)	−1.741	0.082
PFO Types			9.560	0.017
SUT	21 (20.2)	0 (0.0)		
GUT	44 (42.3)	11 (55.0)		
Right funnelform	16 (15.4)	7 (35.0)		
Left funnelform	23 (22.1)	2 (10.0)		
IAS mobility distance	3.00 (2.00, 5.00)	5.00(3.00, 7.00)	−2.547	0.011
PFO angle	20.17 ± 7.22	20.70 ± 7.03	0.300	0.765
RoEP score	6.07 ± 1.95	5.55 ± 1.85	1.097	0.275

### Analysis of factors affecting c-TTE RLS grading in Valsalva manoeuvre

#### Comparison of clinical data of c-TTE RLS classification between two groups

Comparison of clinical data of c-TTE RLS classification between two groups are shown in Table 6. There was a statistically significant difference in the number of PFO morphological cases (*P* < 0.05).

**Table 6:** Comparison of clinical data of c-TTE RLS grading between two groups

Characteristics	c-TTE RLS classification		*t/Z/χ*^*2*^	*P*
	RLS < 2 (*n* = 33)	RLS ≥ 2 (*n* = 91)		
Gender(Male:Female)	20:13	38:53	3.456	0.063
Age	49.61 ± 13.37	49.30 ± 13.37	0.114	0.910
BMI	24.00 ± 3.45	23.51 ± 3.34	0.715	0.476
Left ventricular EF% value	69.09 ± 4.18	70.02 ± 4.25	1.083	0.281
Hypertension	11(33.3)	25 (27.5)	0.404	0.525
Diabetes	2(6.1)	8 (8.8)	0.014	0.904
Hyperlipidemia	9(27.3)	24 (26.4)	0.010	0.920
Unexplained stroke	7(21.2)	17 (18.7)	0.099	0.753
Smoking	9(27.3)	12 (13.2)	3.416	0.065
Drinking	1(3.0)	6 (6.6)	0.102	0.749
Migraine	8(24.2)	32 (35.2)	1.322	0.250
Dizziness/syncope	15 (45.5)	35 (38.5)	0.492	0.483
Chest tightness	3 (9.1)	7 (7.7)	0.000	1.000
Right atrial height of the PFO	1.20 (1.00, 1.50)	2.70 (1.60, 3.30)	−6.073	< 0.001
Left atrial height of the PFO	1.30 (1.10, 1.60)	2.60 (1.50, 3.00)	−3.896	< 0.001
PFO tunnel length	9.00 (6.75, 10.85)	7.80 (6.00, 9.10)	−1.894	0.058
septum secundum thickness	5.80 (4.65, 10.15)	4.30 (3.60, 5.30)	−4.196	< 0.001
PFO Types			26.785	< 0.001
SUT	15 (45.5)	6 (6.6)		
GUT	8 (24.2)	47 (51.6)		
Right funnelform	4 (12.1)	19 (20.9)		
Left funnelform	6 (18.2)	19 (20.9)		
IAS mobility distance	2.00 (1.00, 2.00)	5.00 (3.00, 6.00)	−6.547	< 0.001
PFO angle	18.76 ± 7.08	20.80 ± 7.16	1.410	0.161
RoEP score	5.79 ± 1.73	6.06 ± 2.01	0.678	0.499

The Mann–Whitney *U* test results showed that: the right atrial height of the PFO, left atrial height of the PFO, and IAS mobility distance in the c-TTE RLS ≥ 2 group were significantly higher than those in the c-TTE RLS < 2 group, and the septum secundum thickness was significantly lower than that in the c-TTE RLS < 2 group. There was no significant difference in the PFO tunnel length between the two groups (*P* > 0.05).

#### Logistic regression analysis of the influence of c-TTE RLS classification

Logistic regression analysis of the influence of c-TTE RLS classification are shown in Table 7. The subjects were instructed to perform Valsalva maneuvers, and a binary logistic regression model was established with c-TTE RLS grade ≥ 2 as the dependent variable, and IAS mobility distance and PFO morphology as independent variables. The results showed that the probability of c-TTE RLS ≥ 2 for the SUT PFO was 2.392 times that of the GUT, one unit increase in IAS mobility increased the probability of c-TTE RLS ≥ 2 by a factor of 4.244.

**Table 7:** Logistic regression analysis of the influence of c-TTE RLS classification

Factors	*B*	*SE*	*Waldχ*^*2*^	*P*	*OR* (95% *CI*)
IAS mobility distance	0.872	0.198	19.429	0.000	2.392(1.623 ~ 3.526)
PFO Types	–	–	5.470	0.140	–
GUT	–	–	–	–	1.000
SUT	1.445	0.686	4.444	0.035	4.244(1.107 ~ 16.270)
Right funnelform	1.576	0.866	3.314	0.069	4.835(0.886 ~ 26.381)
Left funnelform	0.789	0.816	0.936	0.333	2.202(0.445 ~ 10.899)
Constant	−2.502	0.652	14.742	0.000	–

#### Diagnostic value of the model for c-TTE RLS classification in Valsalva

**Fig. 7:**
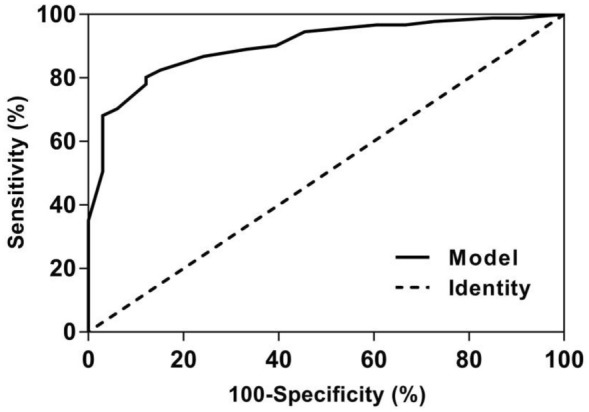
ROC curve analyses of anatomic features of PFO in predicting c-TTE RLS grades

ROC curve analyses of anatomic features of PFO in predicting c-TCD RLS are provided in Fig. 7. The ROC curve showed that the area under the curve for the model to diagnose c-TTE RLS ≥ 2 grade was 0.903 (0.848 ~ 0.958), the corresponding threshold was 0.70287, the sensitivity was 80.2%, and the specificity was 87.9%.

## Discussion

**Fig. 8:**
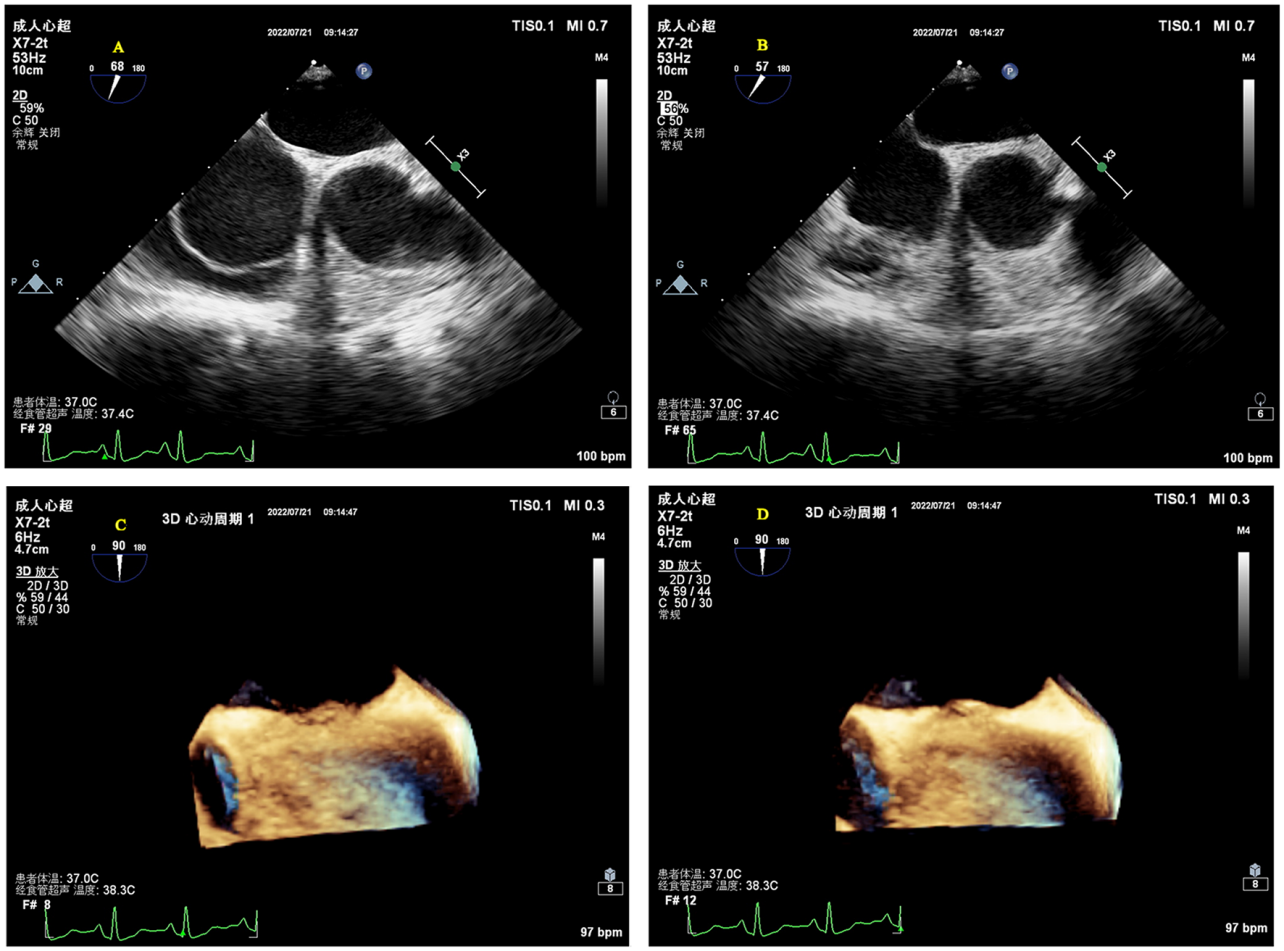
**A** Resting-state GUT (2D TEE). **B** Valsalva-manoeuvre GUT (2D TEE). **C** Resting-state GUT (3D TEE). **D** Valsalva-manoeuvre GUT (3D TEE)

**Fig. 9:**
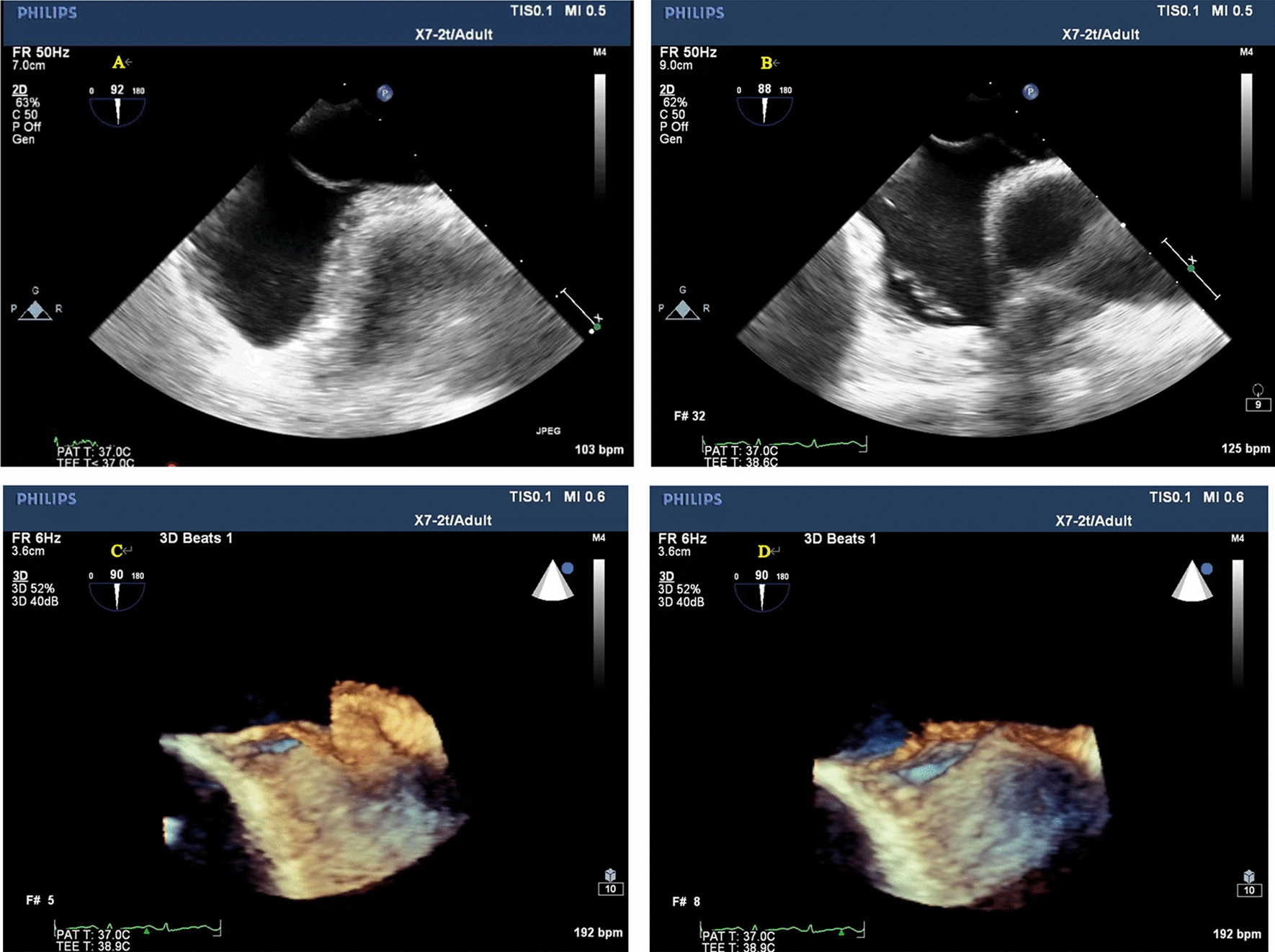
**A** Resting-state right funnelform PFO (2D TEE). **B** Valsalva-manoeuvre Right funnelform PFO (2D TEE). **C** Resting-state Right funnelform PFO (3D TEE). **D** Valsalva-manoeuvre Right funnelform PFO (3D TEE)

Clinically, CS and unexplained migraine in more than half of the patients are often associated with PFO [17]. TTE, c-TTE, c-TCD and TEE are all diagnostic tools for PFO. C-TTE and c-TCD quantitatively evaluate the right-to-left shunt flow of PFO by observing the amount of microbubbles passing through the PFO tunnel, which cannot directly show the characteristics and inner diameter of the PFO tunnel. The study showed that we observed color shunt from the slit-like channel between the septum primum and the septum secundum on TEE, the positive predictive value was 100% [18], which more clearly showed the anatomical structure of the atrial septum, the characteristics of the PFO tunnel and the characteristics of blood flow through the septum. In this study, the anatomical parameters of the PFO observed and measured by TEE were divided into four types, (1) SUT; (2) GUT; (3) Right funnelform; (4) Left funnelform. There are differences in the transseptal blood flow and blood flow characteristics among the four types of PFO: the smooth uniform tubular tunnel PFO transseptal blood flow is uniform in strips, and the granule uniform tubular tunnel PFO transseptal blood flow signal is generally not obvious. Some subjects needed multiple Valsalva maneuvers to display dot-like transseptal blood flow signals. This type of PFO is easy to be missed. The right funnelform and the left funnelform are affected by the degree of right-to-left shunt, and narrow strips of bright blood flow or dim blood flow signals are presented in the tunnel. When subjects were at rest, c-TCD results were divided into RLS ≥ 2 and RLS < 2, there was no significant difference in RLS grades among the four PFO types. After instructing subjects to perform Valsalva maneuvers, the probability of right funnelform PFO RLS ≥ grade 2 was 13.428 times that of GUT, and the specificity was 94.7%. The right funnelform PFO in the resting state has a higher inner diameter of the right atrial side than the left atrial side, and the microbubbles are easier to enter. Mateusz K et al. [19] showed that Valsalva maneuvers increased PFO height and atrial septal offset distance, and increased right-to-left shunt flow. After meta-analysis, PFOs with large shunts had a higher risk of cerebrovascular accident than PFOs with small shunts. This study showed that the structure of GUT PFO is segmental separation of septum primum and septum secundum, and there is no significant change in the right and left atrial height of GUT PFO after Valsalva, as shown in Fig. 8. The right atrial height of the right funnelform PFO increased after subjects underwent Valsalva, as shown in Fig. 9. The c-TTE was divided into two groups: RLS ≥ 2 and RLS < 2. The results showed that the probability of c-TTE shunt ≥ 2 in SUT PFO was 4.244 times higher than that in GUT, with a specificity of 87.9%. The tunnel of SUT PFO is a tubular structure, its blood flow signal is easy to pass through, and the right-to-left shunt flow is large. Therefore, patients with SUT-shaped PFO may be more prone to paradoxical embolism than GUT. However, in this study, the correlation between the four PFOs and stroke showed no significant difference, which may be related to the small sample size, and the sample size needs to be increased for further research. Due to the different RLS levels of the four types of PFO, the volume of ischemic foci that produces paradoxical embolism is also unknown, and further research is needed.

Previous studies have shown atrial septal aneurysm as a risk factor for cerebrovascular accident [20]. Nermin Bayar's study compared the IAS mobility distance between the asymptomatic group and the stroke group, and the IAS mobility distance in the stroke group was significantly higher than that in the asymptomatic group [21], Andre Akhondi MD et al. have confirmed that IAS mobility distance is associated with stroke infarct size, and stroke may also be associated with PFO even in the absence of atrial septal aneurysm [22]. No previous study has demonstrated whether IAS mobility affects the degree of right-to-left shunt. ASA was an exclusion criterion for this study, and the results showed that the probability of c-TCD or c-TTE RLS grade ≥ 2 increased by twofold for each additional 1 mm. It was confirmed that right-to-left shunt is influenced by IAS mobility distance even in the absence of ASA. IAS mobility distance may lead to an increase in right-to-left shunt flow by increasing PFO height. Moreover, with the left and right movement of the atrial septum, the blood of the inferior vena cava may be introduced into the PFO tunnel and thus into the left atrium.

## Study limitations

Due to the small sample size of the study, the current results show that there is no significant difference in the RLS shunt grades between the funnel-shaped and SUT PFO, and the sample size needs to be increased in the later stage for comparison.

PFO samples with Eustachian valve anatomy and Chiari Network were not removed in this study due to the small number of PFO samples with the above structures.

## Conclusions

The four types of PFO have different blood flow characteristics, their anatomical shape and other factors together affect the degree of right-to-left shunt. IAS mobility also increases right-to-left shunt even if the ASA is not present. Observing the specific anatomical characteristics of PFO by TEE can help predict the degree of right-to-left shunt, help to identify the population with high risk of CS, and predict the value of interventional blockade.

## Data Availability

Data and materials could be retrieved from the echo workstation and electronic medical record system in our institution. All data generated or analyzed during this study are included in this published article.
